# Complete remission of a reccurrent mesenteric liposarcoma with rare histological features following the administration of trabectedin

**DOI:** 10.3892/ol.2013.1646

**Published:** 2013-10-29

**Authors:** N. TSOUKALAS, M. TOLIA, G. LYPAS, C. PANOPOULOS, V. BARBOUNIS, G. KOUMAKIS, A. EFREMIDIS

**Affiliations:** 1 2nd Department of Medical Oncology, ‘Agios Savvas’ Anticancer Hospital, Athens, Ampelokipi 11524, Greece; 2 Department of Medical Oncology, ‘401’ General Military Hospital, Athens, Ampelokipi 11524, Greece

**Keywords:** mesenteric liposarcoma, complete remission, trabectedin, Yondelis^®^, dedifferentiation, leiomyosarcoma

## Abstract

The present study describes a rare case of a mesenteric liposarcoma that resulted in a complete remission (CR) following treatment with trabectedin (Yondelis^®^). The patient presented with abdominal pain and fever. An abdominal mass was identified that corresponded to a mixed-type high-grade mesenteric liposarcoma with wide areas of necrosis, areas of dedifferentiation and features of a leiomyosarcoma. Three months after the removal of the first mass, the patient underwent a second laparotomy, followed by treatment with doxorubicin and ifosfamide. Subsequently, the patient was started on therapy with trabectedin and a CR was noted following only four cycles of therapy. The best responses that are reported in the literature for cases of liposarcoma treated with trabectedin are mostly for liposarcomas of the myxoid/round cell type and are mainly partial responses. In the present study, trabectedin was used for the treatment of a mesenteric liposarcoma of mixed morphological features and a CR was achieved.

## Introduction

Soft tissue sarcomas (STS) are rare tumors of mesenchymal origin primarily affecting adults, with liposarcoma being the most frequent histopathological subtype (1,2). Cases of intraperitoneal liposarcoma, particularly primary mesenteric liposarcoma, are rare (3–5). Based on the WHO classification, liposarcoma is classified as well-differentiated (WDL), myxoid, including round/cell liposarcoma, pleomorphic or dedifferentiated (DDL) (6). According to one study, the presence of areas of dedifferentiation in the WDL type occurs in only 7% of cases (7). DDLs are more aggressive than WDLs without dedifferentiation and the rate of recurrence has been reported to be 41% (8).

The present study describes a rare case of a primary sclerosing liposarcoma originating from the root of the mesentery with areas of dedifferentiation and features of a leiomyosarcoma. The patient was treated with trabectedin (Yondelis^®^) and a complete remission (CR) was noted following four cycles of this regimen. Written informed consent was obtained from the patient.

## Case report

**Figure 1:**
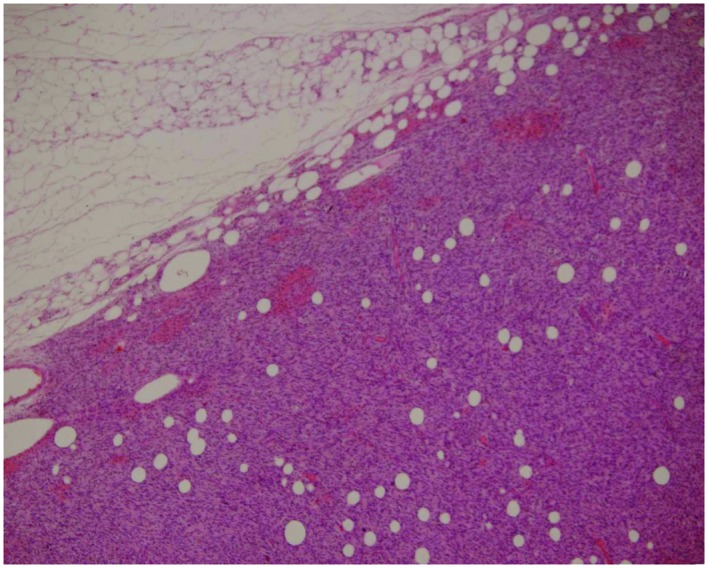
High-grade lobulated sclerosing liposarcoma with focal loss of differentiation. Fatty tissue is present in the upper left area, which contains lipoblasts. In the lower right area, there are areas of dedifferentiation with leiomyosarcoma features. H&E staining; magnification, ×100.

A 47-year-old female with a medical history of hypertension, supraventricular arrhythmia and no clinically relevant family history, presented with abdominal pain and fever. The hematological and biochemical evaluations were within the normal limits. The work-up revealed an abdominal mass. Therefore, the patient underwent a laparotomy five months later. The mass was identified as a liposarcoma originating from the root of the mesentery. The liposarcoma measured ~11.5 cm and was present in the left hypochondrium in contact with the pancreatic tail, but not adhering to the adjacent organs. The pathology report described a high-grade lobulated sclerosing liposarcoma, with focal loss of differentiation and wide areas of necrosis (Fig. 1). The mass measured 15×11×10 cm and was well circumscribed and encapsulated by a thin fibrous capsule. The cut section revealed a fascicular fibrous non-tender tissue of a grey-white color, with the morphological features of a leiomyosarcoma. Immunohistochemistry (IHC) revealed S-100-positive lipoblasts. The liposarcoma was also positive for desmin and smooth muscle actin (SMA), which was consistent with the features of a leiomyosarcoma. Ki-67 staining reactivity was 10% and the liposarcoma was negative for c-Kit (also known as CD117), platelet-derived growth factor receptor and epithelial membrane antigen.

**Figure 2:**
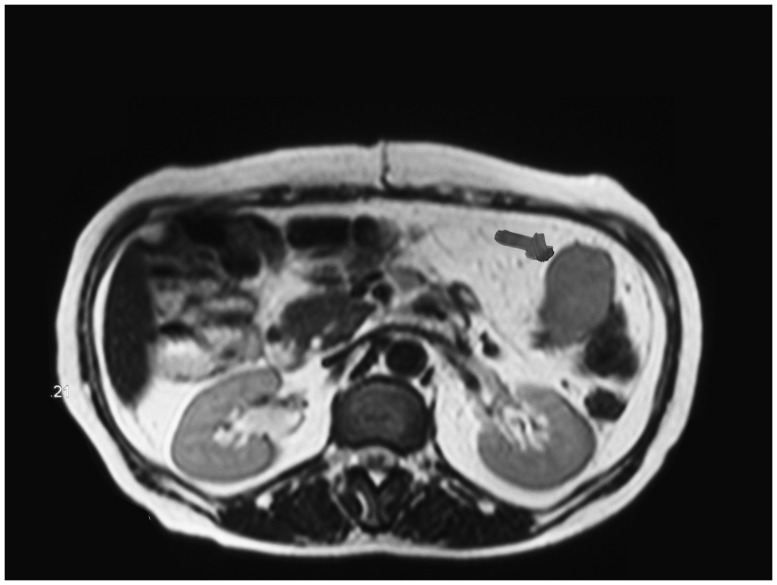
Magnetic resonance imaging (MRI) revealing a local relapse following the completion of three cycles of chemotherapy with doxorubicin and ifosfamide. A T2-weighted MRI axial slice in the middle of kidneys, in which a mass with moderate signal intensity in the left mesenteric area is illustrated.

Three months later, the patient underwent an additional laparotomy to remove a 12×10×3-cm mass of the same histology as the first mass. Consequently, the patient was administered iv chemotherapy with 50 mg/m^2^ doxorubicin and 5 g/m^2^ ifosfamide. The re-evaluation with computed tomography and magnetic resonance imaging (MRI) following the completion of three cycles of chemotherapy revealed a local relapse (Fig. 2).

**Figure 3:**
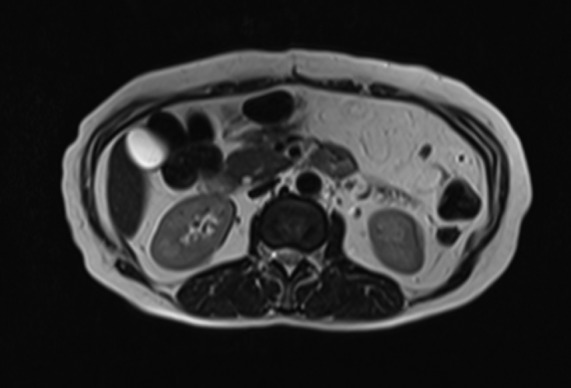
MRI revealing complete remission (CR) according to the RECIST criteria following four cycles of trabectedin. A T2-weighted MRI axial slice in the middle of kidneys, in which the CR of the previous existing mass in the left mesenteric area is illustrated. MRI, magnetic resonance imaging.

Subsequently, the patient was administered trabectedin (1.5 mg/m^2^ in 24 h infusion every 21 days). Following the completion of four cycles, the disease was re-evaluated using MRI, and a complete remission (CR) was noted according to the RECIST criteria (Fig. 3). With regard to the adverse effects and toxicity, the patient complained of diffuse abdominal pain and exhibited grade II vomiting and hepatic toxicity (421 U/l serum glutamic-pyruvic transaminase and 241 U/l serum glutamic oxaloacetic transaminase). Within a few days, the levels of transaminases had returned to normal. The patient continued to experience a CR through 15 cycles of trabectedin therapy.

## Discussion

Cases of primary mesenteric liposarcoma are rare (3–5). The present study describes a case of a mixed-type mesenteric liposarcoma with areas of dedifferentiation and the features of a leiomyosarcoma, including IHC positivity for SMA and desmin. To the best of our knowledge, a case of a mesenteric sclerosing liposarcoma with the morphological and IHC features of a leiomyosarcoma has only been described once (9), while a review of 32 cases of dedifferentiated retroperitoneal and mesenteric liposarcoma included one case of a dedifferentiated mesenteric liposarcoma with smooth muscle elements (5).

Surgical excision represents the cornerstone of treatment for liposarcoma and has been reported to be successful in mesenteric liposarcoma when a clear surgical margin can be achieved (6). Surgery is often followed by radiation and/or adjuvant chemotherapy. However, despite the best locoregional control, disease relapse is common. The standard chemotherapy for the treatment of liposarcoma is doxorubicin combined with ifosfamide (6). Trabectedin has emerged as a favorable option for patients with advanced STSs (10). Trabectedin is a second-line option and is approved for advanced previously-treated STSs in the EU. The drug has shown to be effective in leiomyosarcoma and liposarcoma. Notably, tissue changes have been observed prior to tumor shrinkage (11).

Trabectedin is a marine-derived antineoplastic compound isolated from the Caribbean tunicate *Ecteinascidia turbinata*(12). A modification in the DNA conformation leads to the inhibition of activated transcription, while constitutive transcription appears unaffected (13). Trabectedin binds the DNA minor groove, which, in turn, induces DNA bending towards the major groove. The modification of DNA conformation leads to the inhibition of activated transcription (10). Trabectedin interferes with the transcription factors, DNA binding proteins and DNA repair pathways. Transcription-coupled nucleotide-excision repair appears to be significant in the cytotoxicity of this agent (10). In addition, trabectedin has been shown to modulate the production of cytokines and chemokines by tumor and normal cells, thus altering the microenvironment of the tumor (14).

The antitumor activity of trabectedin has been mainly reported in cases of liposarcoma, particularly the myxoid/round cell type, leiomyosarcomas and synovial sarcomas. The efficacy and safety of trabectedin in a patient population consisting of cases with advanced and/or metastatic liposarcomas or leiomyosarcomas that have failed treatment with standard agents, anthracyclines and ifosfamide, was assessed in a randomized, multicenter, open-label, phase II trial. This randomized clinical trial demonstrated a statistically significant increase in the time to progression and a reduction in the relative risk of progression for patients who were treated with trabectedin (15). Retrospective analyses of the responses in patients with liposarcomas following the failure of anthracyclines and ifosfamide identified the best responses to be mainly partial responses, with CRs occurring most often in myxoid-type liposarcoma cases (16,17). Therefore, the achievement of a CR, as in the present case, appears to occur rarely for liposarcomas of the histological type described in the present study.

Neutropenia, thrombocytopenia, anemia, elevated liver enzymes and increases in bilirubin levels comprise the usual adverse effects of trabectedin. Low grade nausea and vomiting are also frequently encountered. The patient in the present case experienced grade II vomiting and elevated liver enzymes. As in previous studies, these adverse effects were resolved quickly (12,17,18).

In conclusion, the current study presented a rare case of a mixed-type mesenteric liposarcoma. The patient achieved a CR following treatment with trabectedin. It thus appears that trabectedin presents an option for cases of mesenteric liposarcoma with areas of dedifferentiation that have recurred following surgical excision and treatment with standard chemotherapeutic agents, anthracyclines and ifosfamide.
